# Aerosol-jet printing facilitates the rapid prototyping of microfluidic devices with versatile geometries and precise channel functionalization

**DOI:** 10.1016/j.apmt.2020.100618

**Published:** 2020-06

**Authors:** Nordin Ćatić, Laura Wells, Kareem Al Nahas, Michael Smith, Qingshen Jing, Ulrich F. Keyser, Jehangir Cama, Sohini Kar-Narayan

**Affiliations:** a Department of Materials Science and Metallurgy, University of Cambridge, 27 Charles Babbage Road, Cambridge, UK; b Cavendish Laboratory, University of Cambridge, JJ Thomson Avenue, Cambridge CB3 0HE, UK; c Living Systems Institute, University of Exeter, Stocker Road, Exeter EX4 4QD, UK

**Keywords:** Microfluidics, Aerosol jet printing, Lab-on-a-chip

## Abstract

Microfluidics has emerged as a powerful analytical tool for biology and biomedical research, with uses ranging from single-cell phenotyping to drug discovery and medical diagnostics, and only small sample volumes required for testing. The ability to rapidly prototype new designs is hugely beneficial in a research environment, but the high cost, slow turnaround, and wasteful nature of commonly used fabrication techniques, particularly for complex multi-layer geometries, severely impede the development process. In addition, microfluidic channels in most devices currently play a passive role and are typically used to direct flows. The ability to “functionalize” the channels with different materials in precise spatial locations would be a major advantage for a range of applications. This would involve incorporating functional materials directly within the channels that can partake in, guide or facilitate reactions in precisely controlled microenvironments. Here we demonstrate the use of Aerosol Jet Printing (AJP) to rapidly produce bespoke molds for microfluidic devices with a range of different geometries and precise “in-channel” functionalization. We show that such an advanced microscale additive manufacturing method can be used to rapidly design cost-efficient and customized microfluidic devices, with the ability to add functional coatings at specific locations within the microfluidic channels. We demonstrate the functionalization capabilities of our technique by specifically coating a section of a microfluidic channel with polyvinyl alcohol to render it hydrophilic. This versatile microfluidic device prototyping technique will be a powerful aid for biological and bio-medical research in both academic and industrial contexts.

## Highlights

• Aerosol-jet printing is used to fabricate microfluidic devices with customised geometries, including steps and slopes.
• A rapid prototyping method for producing bespoke molds for microfluidic devices with precise channel functionalization.
• The functionalization capability is demonstrated by rendering a section of a microfluidic channel hydrophilic using PVA.

## 1 Introduction

Over the past couple of decades, microfluidics has revolutionized biological and biomedical research by enabling the precise analysis of samples in well controlled conditions while using minimal reagents, and has facilitated a paradigm shift in the way such research is conducted [[1], [2], [3]]. Techniques such as injection molding [4], hot embossing [5] and laser ablation [6] are often used as production methods for microfluidic devices. These processes may be suitable for the high-volume reproduction required for emerging commercial and clinical applications of microfluidics. However, the vast majority of microfluidic devices are used in a laboratory setting for research and development. The ability to rapidly prototype new designs is hugely beneficial in a research environment, yet the high cost, slow turnaround and wasteful nature of the above techniques severely impedes the development process. This is also a challenge within industrial research as rapid prototyping is key during the crucial device optimization process. Delays caused by waiting for masks or molds can cost hundreds of thousands of dollars [7] and also days or weeks in manufacturing, due to the lack of a suitable fast prototyping method.

In this context, *soft lithography* has become the method of choice for research scientists when creating microfluidic devices for research and development purposes [8,9]. In this technique, a pre-polymer is cast over a mold whose surface contains the profile of the desired network of microfluidic channels and junctions. The pre-polymer is then cross-linked, either through a chemical agent or UV radiation, to create a relief of the mold surface. The cross-linked polymer is removed from the mold and bonded to a flat surface, typically a glass coverslip, to seal the channels and complete the microfluidic device.

The master mold is often produced using photolithography [9]. This is a well-established technique, but the time, cost and resources required to produce new designs considerably limits the rate of development. This is particularly relevant when it comes to producing more complex molds in 3D or with changes in height [10]. As a result, additive manufacturing is attracting significant attention as an alternative process through which microfluidic devices can be rapidly prototyped [11]. In some instances, 3D printing is being used to create the entire microfluidic device, without the need for soft lithography [12]. Techniques such as stereolithography (SLA) [12], two-photon polymerization (2PP) [13] and bioplotting [14] are capable of producing complex and intricate designs, but are limited in mass production by slow build-rates, a limited choice of materials and low build volumes [11]. There have been attempts at creating sacrificial molds by lithography [15,16] as well as 3D printing [17,18]. Most of these methods then require that the mold be melted and removed through the channel [[16], [17], [18]] under pressure which can take up to 27 h to fully flush [19].

However, even though current additive manufacturing is sub-optimal for the mass production of complete microfluidic devices, producing molds for soft lithography offers a much faster and more flexible way of prototyping microfluidic designs. Designs can be changed during the production process and made within a few hours. The main challenges involved in this route include rough channel surface topologies and an inability to print channels down to the sizes that photolithography can produce [20]. To this end, inkjet printing has been used to create microfluidic channels by using an ink that instantly freezes when it touches a cooled substrate [19]. After following traditional soft lithography steps with PDMS, the mold is then removed through sublimation such as the lost wax method [21]. This results in a single-use mold with a minimum feature size of 50 μm laterally and 16 μm vertically. Another approach, as described by Coppola et al., shows the use of pyro-electric activated inkjet printing [22,23]. This method resulted in fibers with diameters as small as 10 μm which were then used as molds to create channels. By controlling certain parameters, they propose that it is possible to produce fibers that are 1 μm in diameter. Notably, using this technique it is possible to create 3D microstructures. However, as in the previous method, this technique requires the sacrifice of the mold after encapsulation which limits its use in mass production, as a new mold would need to be printed for every device.

**Fig. 1:**
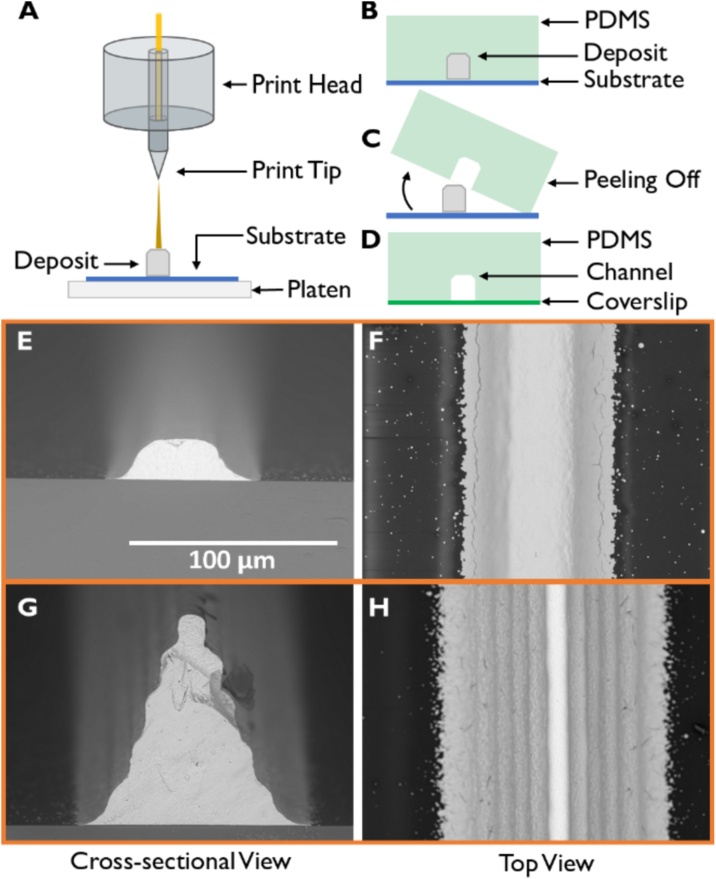
Schematic of the device manufacturing process starting with the printing of a mold, followed by soft lithography as well as SEM images of printed molds. **(A)** The Aerosol Jet Printer works by atomizing ink which is picked up by the nitrogen carrier gas. This ink loaded nitrogen is then fed into the print head and jetted from the print tip onto the substrate creating a mold. Inside the print head, a sheath gas of nitrogen surrounds the ink loaded nitrogen gas which further focuses the deposition area. The platen underneath the substrate can be heated to change the wetting and adhesion properties of the ink on the substrate. **(B)** After curing the mold, PDMS (a 10:1 ratio of elastomer to curing agent) is poured over it and cured until hardened. **(C)** Once the PDMS has hardened, the substrate and mold are peeled away leaving a channel inside the PDMS. The PDMS is then rinsed and dried. **(D)** The PDMS chip has inlet and outlet holes punched following which it is cleaned and plasma bonded onto a coverslip. **(E)** Cross-sectional SEM image of a silver mold printed onto a PVA coated glass slide. **(F)** Top down SEM image of the silver mold in E. **(G)** Cross-sectional SEM image of a triangular silver mold design printed onto a PVA coated glass slide, where the single printed line at the top of the triangular mold has a width of ∼10 μm. **(H)** Top down SEM image of the silver mold in G. Scale bar is the same for E–H.

Here, we demonstrate the use of Aerosol-Jet Printing (AJP) [24] to produce molds for the soft lithography of microfluidic devices (Fig. 1; an image of the actual printer used is also shown in Supplementary Fig. 1). The resolution of the printing technique allows features down to 10 μm laterally [24] and 0.5 μm vertically to be fabricated over a large area (15 × 15 cm^2^) at a fraction of the cost, time and resources required by photolithography. Our technique differs from the recent work of Di Novo et al., who also used an AJP to make microfluidic devices by printing a material that can be UV cured instantly after leaving the tip [25]. Their method directly produces the channels and not a mold, and is prone to a deviation of 8% between prints [25]. The molds produced using our technology report a 5× improvement in lateral resolution and a 32× improvement in vertical resolution over standard inkjet printing methods [19]. Compared to devices produced recently using pyro-electric activated inkjet printing, devices produced using our AJP show similar lateral resolution and a 20× improvement in the vertical resolution; however, we note that it has been proposed that the pyro-electric activated inkjet technique can develop fibers with diameters down to 1 μm, for use as molds [23].

**Fig. 2:**
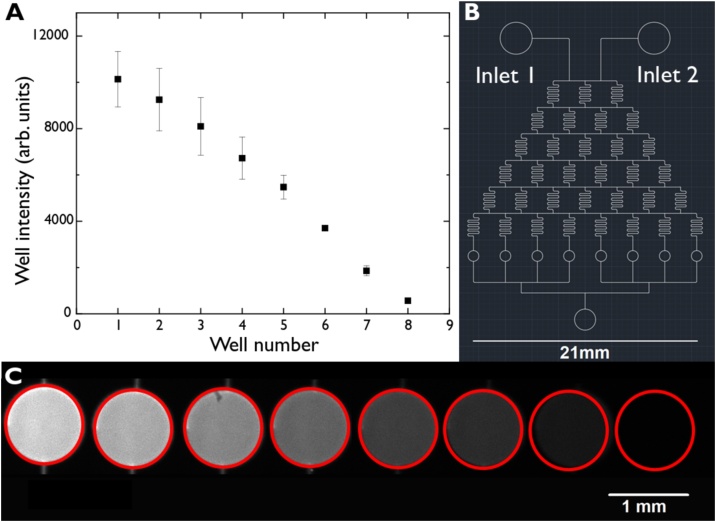
**(A)** Average fluorescence intensity of each well (mean ± std. dev. for 2 repeats) showing the input mixing gradient of a fluorescent dye across all 8 reservoirs. This was produced by having a mixing device (schematic shown in **(B)**) that has two inlets where fluorescent dye and a buffer are injected. The two solutions are pumped through at 1 bar and are continuously split at T-intersections to create a gradient of the fluorescent solute concentration within the serpentine sections. The solutions are then sent into wells at the end of the device for imaging. The images from the wells are shown in **(C)**. They show the expected intensity gradient across the device where the left most well has only fluorescent dye and the right most well has only buffer solution.

To showcase how inexpensive AJP fabricated molds can be, we note that the material cost to print the mixing design in Fig. 2 was only a fraction of a cent. In contrast, the cost of ordering a mask for photolithography can be significant (see Supplementary Information Table 1) depending on the base material, the size of the mask, and the turnaround time required. This does not take into account the cost of the photoresist material which itself is fairly expensive and mostly wasted during the photolithography process; note this applies even when photolithography is performed using direct laser writing, where the need for a mask is obviated. Due to the distance between the tip and the substrate, the AJP method can print on almost any substrate even if it is not flat and uses minimal amounts of material to make a mold. The material used goes directly into making the mold and is not wasted at any step during the process.

Importantly, like other additive manufacturing techniques, the AJP method facilitates structures and geometries that are either impossible or exceptionally challenging with photolithography. We can also take advantage of the broad range of materials that can be deposited with AJP to produce microfluidic channels with micro-patterned coatings, which facilitates the precise functionalization of the microfluidic device.

Photolithography via direct laser writing is an increasingly commonly used method for making molds for microfluidic devices. It is faster than mask-based photolithography and has an acceptable resolution when prototyping new designs. When compared to AJP, the initial equipment cost is relatively similar and in terms of time-scale to produce research prototypes they are almost identical. However, AJP can print over larger areas than standard direct laser write systems, does not waste any material, and can add slopes and functionalize the channels as well; there are thus major advantages of using AJP over conventional photolithographic methods for producing microfluidic molds.

## 2 Results and discussion

Aerosol jet printing may be performed using either organic or inorganic materials. Fig. 1 shows the device manufacturing process involved in making a mold using an AJP. The “ink”, i.e., the material used to construct the mold, is atomized into an aerosol through either an ultrasonic or a pneumatic atomizer, depending on the ink parameters. This ink aerosol is transported to the deposition head by a carrier gas (nitrogen) and jetted onto the substrate as shown in Fig. 1(A). The deposited ink thus forms the mold. Once the mold has been created and cured, standard soft lithography protocols [8,26] are followed to create and assemble the microfluidic devices. We achieved molds with widths down to ∼10 μm with a minimum feature height of 0.5 μm. Fig. 1(E) and (F) show a channel designed to form a square profile. Through changes in printing parameters, the channel designs can be made to have a more circular profile or even profiles that have a triangular cross section such as the one depicted in Fig. 1(G) and (H). The widths and heights of straight line molds were checked on a Dektak profilometer before and after curing. The curing process results in a significant, but relatively constant, change in the height of the molds, whereas the width shows a smaller change, on the order of 1% of the original width (Supplementary Information Table 3). To be more indicative of the actual channel, dimensions are taken from molds after curing for descriptive purposes in this paper.

A simple fluid mixer (gradient generator) was printed to show that devices using an AJP are comparable to those fabricated using other techniques. The mixing design is shown in Fig. 2. The left inlet was pumped with a fluorescent dye while the right inlet contained a non-fluorescent buffer solution. Flows were controlled using a pressure driven pump. The solutions split equally at each T-junction and are mixed within the serpentine section before being split again. This was repeated until 8 reservoirs were filled with solution where the left-most reservoir had only fluorescent solution, the right-most reservoir only had buffer solution, and the 6 in between displayed a uniform fluorescence gradient across them (Fig. 2). This result shows that uniform laminar flows were achieved (see Supplementary Fig. 2) at the T-junctions while diffusion mediated mixing was achieved to create a concentration gradient of the fluorophore within the serpentine section. Silver as well as Polyimide (PI) molds were tested for this design and both exhibited uniform laminar flow. These tests were performed immediately following the soft lithography and bonding protocols to create the microfluidic device.

**Fig. 3:**
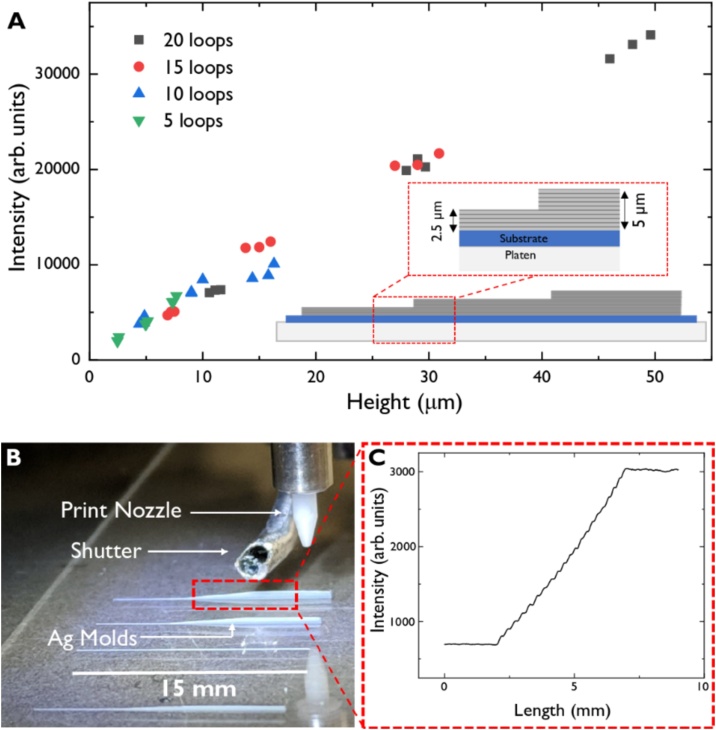
**(A)** Channels with two steps were made and the light intensity at each step was measured. Four different channels were made with steps of different sizes and a fluorescent dye was pumped through. The plot compares the measured channel heights to the intensity of light recorded by a fluorescence microscope, which shows a clear linear trend as expected. The diagram at the bottom right is based on a ‘5 loop’ system. The first layer of 5 loops is printed with each loop depositing ink with a height of 0.5 μm. This results in the base layer having a height of 2.5 μm. A second layer of 5 loops is printed with shortened lines. This results in another layer of 2.5 μm which in combination with the base layer has a total height of 5 um. Another layer is then printed in the same way to create 3 layers on top of each other leading to two steps each having a 2.5 μm change in height. **(B)** Image of molds with different slopes being printed. **(C)** Plot showing the intensity of light due to a fluorescent dye being pumped through a sloped channel. By having the same channel width throughout, the change in intensity corresponds to a change in height of the channel. This was done in a similar way as in (A) but by having fewer loops at each step and making them closer together leading to a smoother increase in height.

One of the major differences between the current standard methods of microfluidic manufacturing and our method is the ease of making steps and slopes within the device. A step is defined as a change in height within a line. This is created by printing a base layer followed by a second layer that is shorter than the previous one. The size of the step can be changed by altering the height of each layer. This is done by changing the number of times the printer goes over each layer, also referred to as the number of loops. This can be visualised in the diagram in Fig. 3(A). Two steps were created by having a base layer with 5 loops where each loop deposits ink with a height of 0.5 μm creating a total height of 2.5 μm for the base layer. Printing another shorter layer on top of the base layer leads to a step of 2.5 μm.

Four different channels with 2 steps in each were made. These channels differed by changing the number of loops at each step (5, 10, 15, 20). Fig. 3(A) shows the change in intensity measured by the fluorescence microscope, for a fluorescent dye pumped through the device, and compares it to the height of the channels (see Experimental Section). As the steps all have similar widths, the fluorescence intensity linearly correlates with the channel height. Three channels were printed for each of the loops and the height was measured at each layer.

With a vertical resolution of 0.5 μm per printed line, it is possible to modify the printing designs to create steps and slopes with high accuracy. The slope is made by printing successive lines of decreasing length directly on top of each other, leading to many micro-steps that form a slope. Fig. 3(B) shows a range of molds containing varying degrees of slopes. The smoothness and angle of the slope can be changed by making the steps further apart or closer together. The minimum length change between each step is 5 μm due to the machine tolerance involved in moving the platen. The distinct steps are clearly characterised when a fluorescent dye is pumped through a sloped device, as shown in Fig. 3(C) where 20 steps are printed close to each other to create a slope. This makes it easy to create a device where having varying channel heights throughout the device is necessary [10,27].

Various applications [28,29] require only specific areas of the channel network to be coated, which is technically challenging using standard methods. However, by directly printing coating materials on the required sections of the channel network, our technique enables us to circumvent this challenge in a fast and highly reproducible manner. As an example, T-intersection molds were printed using polyimide followed by the selective printing of polyvinyl alcohol (PVA) on a defined section of the molds. The inlet and one of the sides of the intersection were coated leaving the other side without any PVA to test whether the selective PVA coating could affect the direction of fluid flow within the microfluidic device. We chose PVA since it is commonly used as a coating to render channels hydrophilic [29].

In our test, once the PVA dried, PDMS was poured over the mold and left to cure. When the PDMS was peeled off, the PVA adhered to the PDMS device and the PI mold was left on the glass substrate.

**Fig. 4:**
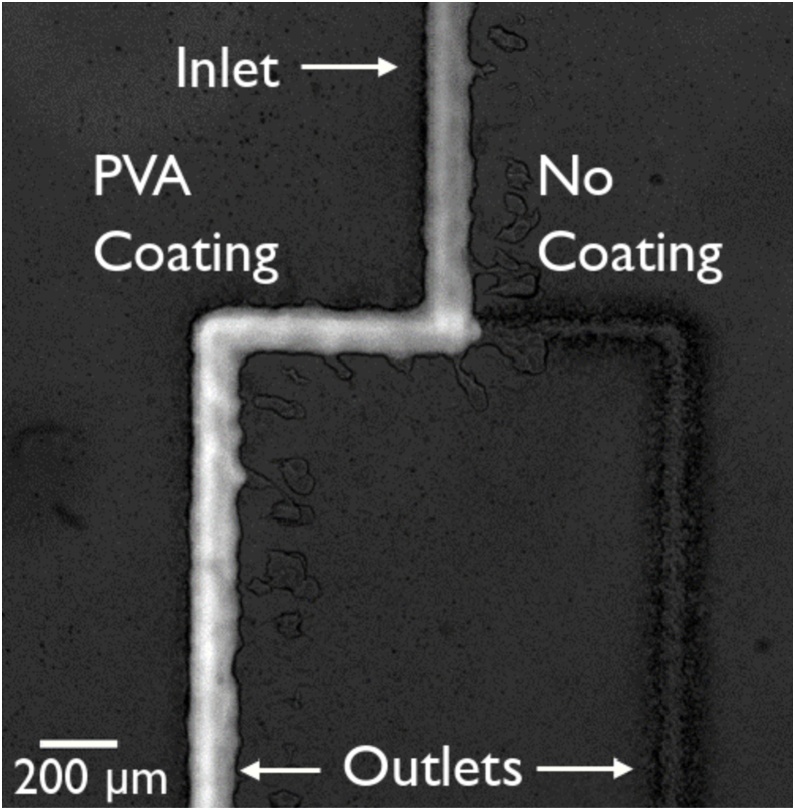
Fluorescent dye in an aqueous solution passing through a PVA coated channel. The fluid enters via the inlet (at the top of the image) and is split at the T-intersection. All the liquid preferentially goes towards the side containing the PVA coating while none of it goes towards the non-coated side.

The PDMS chip was plasma bonded to a glass slide and left at room temperature for 24 h to ensure that the effects of the plasma treatment on PDMS would not affect the results. HPTS (Pyranine) fluorescent dye was passed through the inlet at the top of Fig. 4 and can be seen flowing only to the left, where the PVA coating was printed (a schematic explanation of the PVA deposition process is shown in Supplementary Fig. 3). The slight widening observed in the image of the fluorescent dye passing through the PVA-coated channel was due to residual PVA that was transferred onto the glass and extended beyond the channel dimension (shown in Supplementary Fig. 4). This shows that the PVA coating fully transferred during peel off and can successfully direct fluid flow within the device. The same results were seen when PVA was only printed after the intersection point and the inlet was left PVA-free. This method requires PVA to be re-printed each time for each set of devices, giving the user flexibility on the placement of the coating as the same mold can be reused with PVA deposited in different locations if desired. We note that the PVA coated channels studied here have a nominal height of 15.3 ± 0.5 μm (mean ± s.d. for 5 samples).

## 3 Conclusions

Various methods to manufacture microfluidic devices have been described and compared to the current industry accepted method of photolithography. Compared to other additive manufacturing techniques such as regular inkjet printing that report a minimum feature size of 50 μm laterally and 16 μm vertically [19,30], we show the successful fabrication of microfluidic devices using aerosol jet printing with at least a 5× improvement in X–Y resolution and a 32× improvement in Z resolution [11,31]. Pyro-electric activated inkjet printing appears to have better resolution in the lateral dimension and a similar resolution vertically [23]. However, unlike our molds, this method requires the sacrifice of the mold material and thus provides single-use molds only.

The ability to print and cure a new mold in less than 3 h and re-use it multiple times will make the prototyping of new designs much more efficient than currently feasible. As well as creating a fast prototyping method, this technique has also facilitated the convenient integration of geometries such as steps and slopes We have also demonstrated that passive coatings can easily be printed onto specific portions of the channel network to aid in controlling the flow of fluids. In a similar way, passive protective coatings may potentially also be printed to enable the use of solvents that would otherwise cause swelling and degradation of the microfluidic devices.

Thus, aerosol jet printing has the potential to revolutionize microfluidic device manufacture by prototyping at shorter timescales, with minimal material costs, producing versatile geometries with the added ability of introducing new functionality directly and specifically within the channels. These novel capabilities will greatly expand the scope of microfluidic technologies in chemical, biological and biomedical research.

## 4 Experimental section

### 4.1 Materials

Two different inks were used in this study to make molds: silver and polyimide. The silver was made using Clariant Silver Nanoparticle Ink (Clariant Prelect TPS 50G2) mixed in a 1:1 ratio with deionised water. The polyimide ink was made by mixing Polyamic acid stock solution (12.8 wt.% polyamic acid in 80% NMP/20% aromatic hydrocarbons, Sigma Aldrich) in *N*-Methyl-2-Pyrrolidone (NMP) in a 1:1 ratio.

The PVA was made by dissolving PVA (87–90% hydrolysed, molecular weight 30,000–80,000 Da) in deionised water at 2.5 wt.%.

### 4.2 Aerosol jet printing

Using an Optomec Aerosol Jet 200 Printer, the inks were atomised for printing through either an ultrasonication or a pneumatic method [24]. The silver ink was printed using an ultrasonic atomiser while polyimide was printed using a pneumatic atomiser. In both cases, an inert nitrogen gas was used to transport the atomised ink to the nozzle and jet onto the substrate. A regular glass slide was used as a substrate when printing polyimide while a PVA coated glass slide was used for silver to aid with adhesion.

### 4.3 Mold fabrication and coatings

The molds were designed using AutoCAD and printed using the Optomec Aerosol Jet Printer AJ200. Using a raster pattern, the width of the mold can be made as small as 10 μm with a minimum height of 0.5 μm. The height is controlled by changing either the number of loops of the raster pattern or the speed of the printing. When the molds are printed, they are cured in an oven to make sure that any residual solvent is evaporated away. Silver ink was cured at 150 °C for 2 h and polyimide was cured at 200 °C for 2 h. The PVA coating was printed using a pneumatic atomiser and ethanol was added to prevent the ink from foaming in the atomiser (see Supplementary Information Table 2 for printing parameters).

### 4.4 Device fabrication

To make the devices, Polydimethylsiloxane (PDMS, Sylgard 184, Dowsil) was prepared in a 10:1 ratio of elastomer to curing agent. The mixture was degassed under vacuum, poured over the molds and cured in an oven for 2 h at 80 °C. After cooling the PDMS to room temperature, it was cut and peeled off the molds and checked under a microscope to ensure that none of the mold had broken off and adhered to the PDMS. Of the materials tested, the only mold that was seen to leave particles in the PDMS channels was silver. On such occasions, the silver was etched away in a ferric nitrate solution (0.2 g/ml of water) at room temperature for 24 h.

Once the PDMS devices were cleared of particles, they were further rinsed in ethanol to remove any residual particles from the handling or the cutting process. Inlet and outlet holes were created by using biopsy punches with diameters ranging from 0.75 mm to 3 mm depending on downstream testing. The final devices were assembled using a standard plasma bonding protocol for bonding PDMS onto glass slides [9,28].

### 4.5 Optical setup and flow control

The fabricated microfluidic devices were visualized using an Olympus IX73 inverted microscope. The microscope is equipped with a 4× air objective (UPlanFLN Olympus, NA 0.13), a 10× air objective (UPLFLN Olympus, NA 0.3), a 20× air objective (UPlanFL Olympus, NA 0.7) and a 60× water immersion objective (UPLSAPO Olympus, NA 1.2). Images were acquired using a Photometrics Evolve 512 camera controlled via μManager 1.4 software [32] with a wLS LED lamp from QImaging as the light source during fluorescence measurements. A FITC filter cube set (Chroma) was used for tracking HPTS (Pyranine) fluorescence at a concentration of 50 μM. For the fluorescence imaging of HPTS in the various channel geometries studied, the LED intensity was 10% with a camera exposure time of 10 ms and an EM gain of 100.

The microfluidic flows were controlled using a pressure driven pump; we used the MFCS-EZ™ (Fluigent GmbH, Germany) flow control system with its accompanying MAESFLO™ software (version 3.2.1). The fluid reservoirs (Micrewtube 0.5 mL, Simport) were connected to the microfluidic chip via a polymer tubing (Tygon microbore tubing, 0.020″ID × 0.060″ OD, Cole Parmer, UK) and a metal connector tip. Dispensing tips (Gauge 23 blunt end, Intertronics) served as connector tips between the tubing and the chip. The fluids in the chip were controlled using 2 separate pressure ports operating at 1 bar.

## Declaration of competing interests

The authors have no competing interests to declare.
