# Supplementary material for: Aerosol-jet printing facilitates the rapid prototyping of microfluidic devices with versatile geometries and precise channel functionalization
Source: Appl Mater Today. 2020 Jun;19:100618. doi: 10.1016/j.apmt.2020.100618 (PMC7821597; doi:10.1016/j.apmt.2020.100618)
Supplement: Supplementary file 1 [file mmc1.pdf]

Supporting Information

Aerosol-jet printing facilitates the rapid prototyping of microfluidic devices with versatile geometries and precise channel functionalization

Nordin Ćatić,[a] Laura Wells,[a] Kareem Al Nahas,[b] Michael Smith,[a] Qingshen Jing,[a] Ulrich F. Keyser,[b] Jehangir Cama,[b,c,]* and Sohini Kar-Narayan[a,]*

[a]*Department of Materials Science and Metallurgy, University of Cambridge, 27 Charles Babbage Road, Cambridge, UK.*

[b]*Cavendish Laboratory, University of Cambridge, JJ Thomson Avenue, Cambridge CB3 0HE, UK.*

[c]*Living Systems Institute, University of Exeter, Stocker Road, Exeter EX4 4QD, UK*

Correspondence: sk568@cam.ac.uk, j.cama@exeter.ac.uk

Supplementary Table 1*:* Comparing Aerosol Jet Printing with other techniques commonly used for making microfluidic devices based on resolution, cost, time and hazards associated with each technique. (References cited from main text)

	**Aerosol Jet Printer**	**Mask-based Photolithography**	**Laser Writer**	**3D Printer**	**Pyro-electrospinning inkjet printing**
**XY resolution**	10 µm[24]	0.5-1µm	1µm	50 µm[30]	10 µm[1][23]
**Z resolution**	0.5 µm	< 1 µm	< 1 µm	20 µm[30]	10 µm[23]
**Material cost per mold**	0.5¢ -5¢	>£10	>£10	£1-£5	Unknown
**Time**	Hours	Days	Hours	Hours	Hours
**Hazards**	Nano particulates	Ultraviolet exposure, photoresist chemicals	Ultraviolet exposure, photoresist chemicals.	Toxic/Carcinogenic (depending on print material)	Large Voltage[22]

[1] Fiber diameters were demonstrated between 10 and 30 µm though it is proposed that smaller dimensions down to 1 µm can be achieved.

Supplementary Table 2. Comparison between print parameters when using silver and polyimide ink.

	**Silver**	**Polyimide**
XY resolution	10µm ±1	120±2
Z resolution	0.5µm ± 0.2	0.5µm ± 0.2
Curing temperature	150°C	200°C
Curing time	2 hours	2 hours
Atomizer	Ultrasonic	Pneumatic
Reusable	No	10+
Tip Size	150 µm	200 µm
Print Parameter	S: 60<br>Atm: 28 ± 2	S: 80<br>Atm: 950 ± 100<br>Ex:900 ± 100
Hazards	Nanoparticles	Toxic Solvent

Supplementary Table 3. Table showing the effects of curing on the width and height of polyimide channels.

	Pre-Curing		Post-Curing		Change	
**Sample**	**Width (µm)**	**Height (µm)**	**Width (µm)**	**Height (µm)**	**Width (%)**	**Height (%)**
**1**	200	40	192	26.2	-4.0	-34.5
**2**	200	40	198	26.2	-1.0	-34.5
**3**	200	40	193	25.9	-3.5	-35.3
**4**	198	40	208	25.9	5.1	-35.3
**5**	198	40	193	26.2	-2.5	-34.5
**6**	196	40	195	26.4	-0.5	-34.0
**7**	199	40	198	26.3	-0.5	-34.3
**8**	195	39.6	199	26.2	2.1	-33.8
**9**	196	40.7	192	26.5	-2.0	-34.9
**Average**	**198.00**	**40.03**	**196.44**	**26.20**	**-0.8**	**-34.6**
**STDEV**	**1.94**	**0.28**	**5.13**	**0.20**	**2.8**	**0.5**

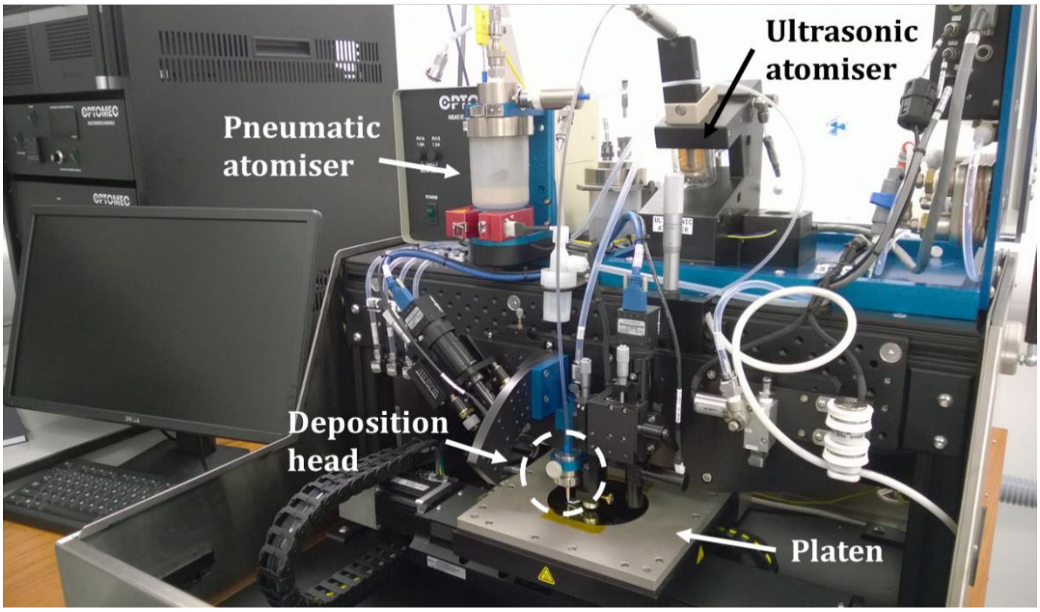

Supplementary Figure 1: Image of the Aerosol Jet Printer showing the locations of the ultrasonic and pneumatic atomisers as well as the connections to the deposition head. The printer used is an AJ200 from Optomec (Optomec Inc. New Mexico, USA)

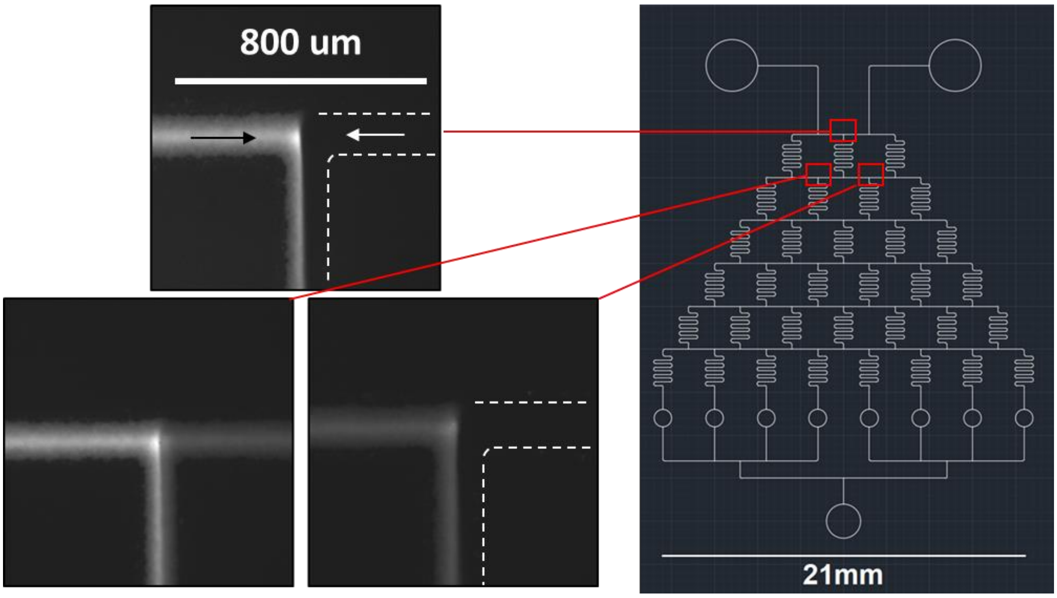

Supplementary Figure 2: **(Left)** Images showing the laminar flow of fluorescent and non-fluorescent solutions at the T-intersections of the mixing devices, used to create a concentration gradient of the fluorophore in the channel network. **(Right)** schematic of the mixing device showing the location of the T-junctions corresponding to the microscopy images on the left. The microscopy images show that the flows at the T-junctions are laminar, with no turbulent mixing, as expected. The fluorescence imaging settings are described in the Experimental Section.

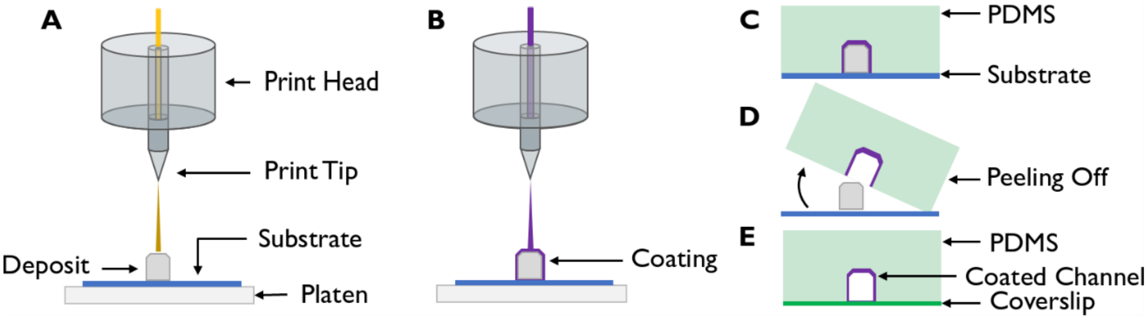

Supplementary Figure 3. Schematic of the process of adding a coating to the channels starting with the printing of a mold, followed by deposition of the coating material on the mold, and completed by soft lithography. (A) The Aerosol Jet Printer works by atomizing ink which is picked up by the nitrogen carrier gas. This ink loaded nitrogen is then fed into the print head and jetted from the print tip onto the substrate creating a mold. Inside the print head, a sheath gas of nitrogen surrounds the ink loaded nitrogen gas which further focuses the deposition area. The platen underneath the substrate can be heated to change the wetting and adhesion properties of the ink on the substrate. (B) The mold is cured following which a defined amount of the coating material (we tested PVA) is deposited onto the mold in the desired location. (C) PDMS (a 10:1 ratio of elastomer to curing agent) is poured over the coated mold and cured. (D) Once the PDMS has hardened, the substrate and mold are peeled away leaving the coating adhered to the channel inside the PDMS. (E) The PDMS chip has inlet and outlet holes punched following which it is cleaned, and plasma bonded onto a coverslip.

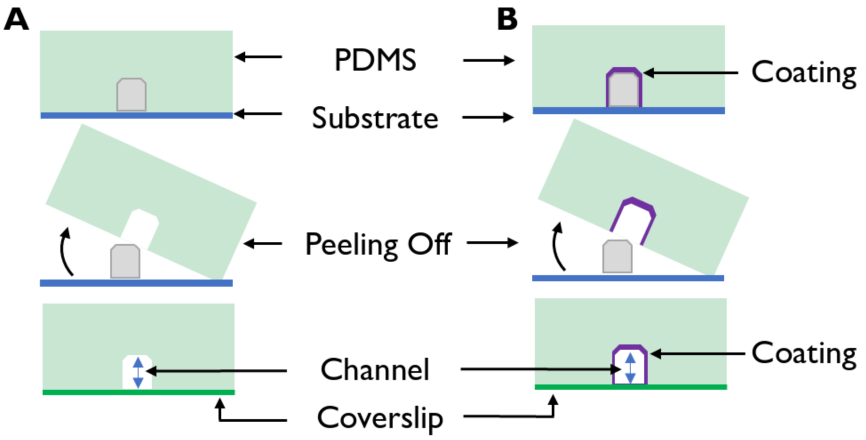

Supplementary Figure 4. *Schematic showing the channel dimensions with and without an additional coating.* ***(A)*** *and* ***(B)*** *have the same channel dimensions with the difference being that the walls of the channel in A are made of PDMS only while in B the coating lines the channel walls.*
